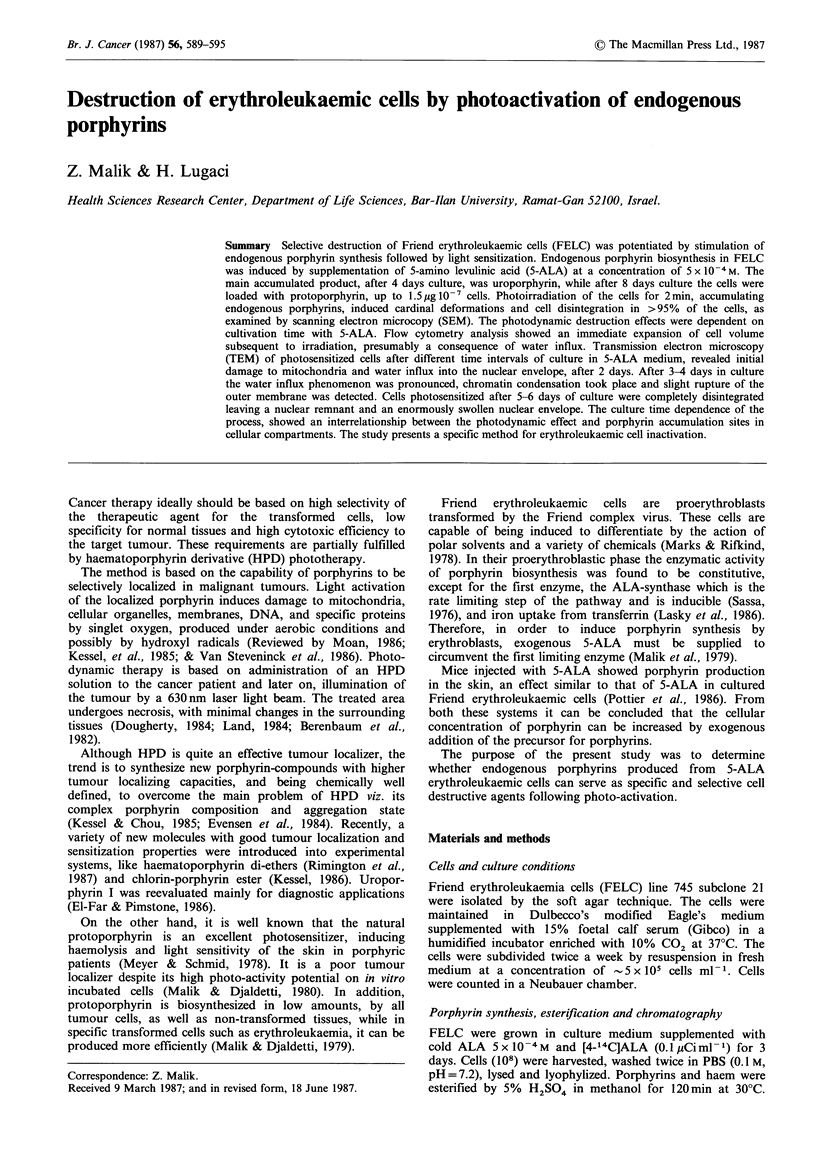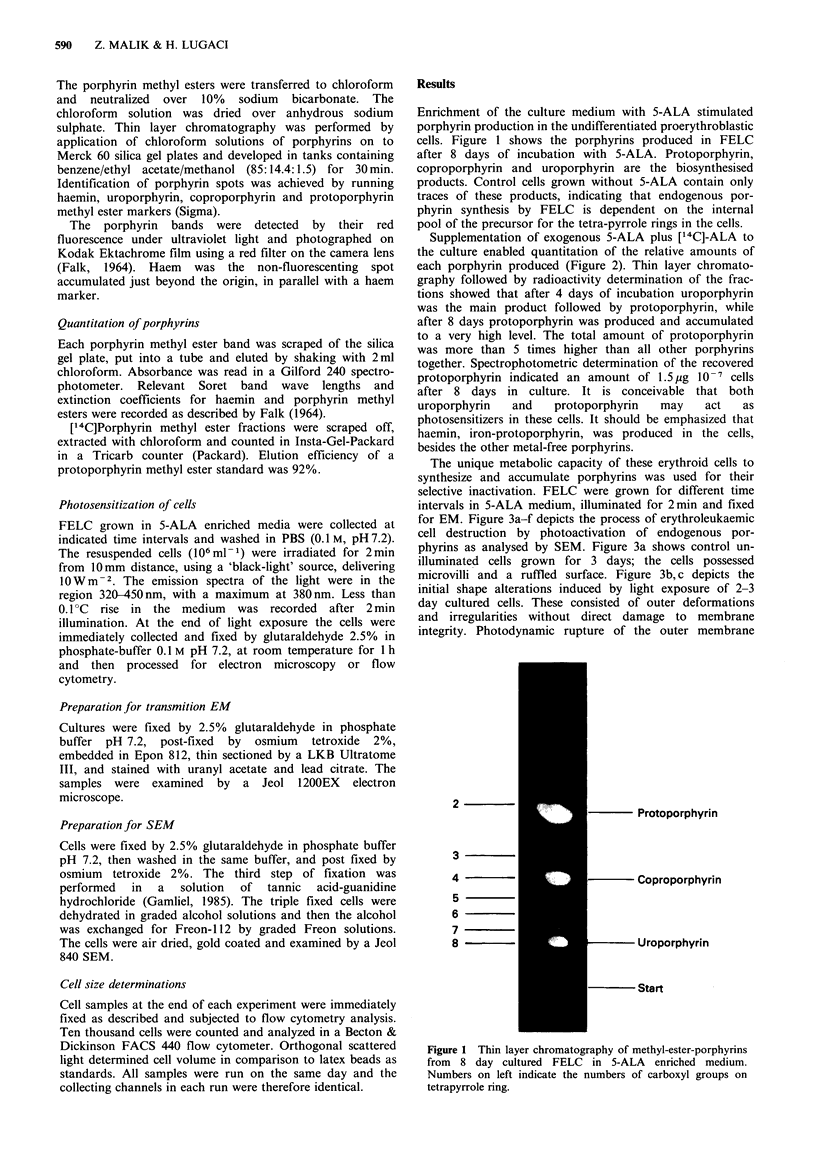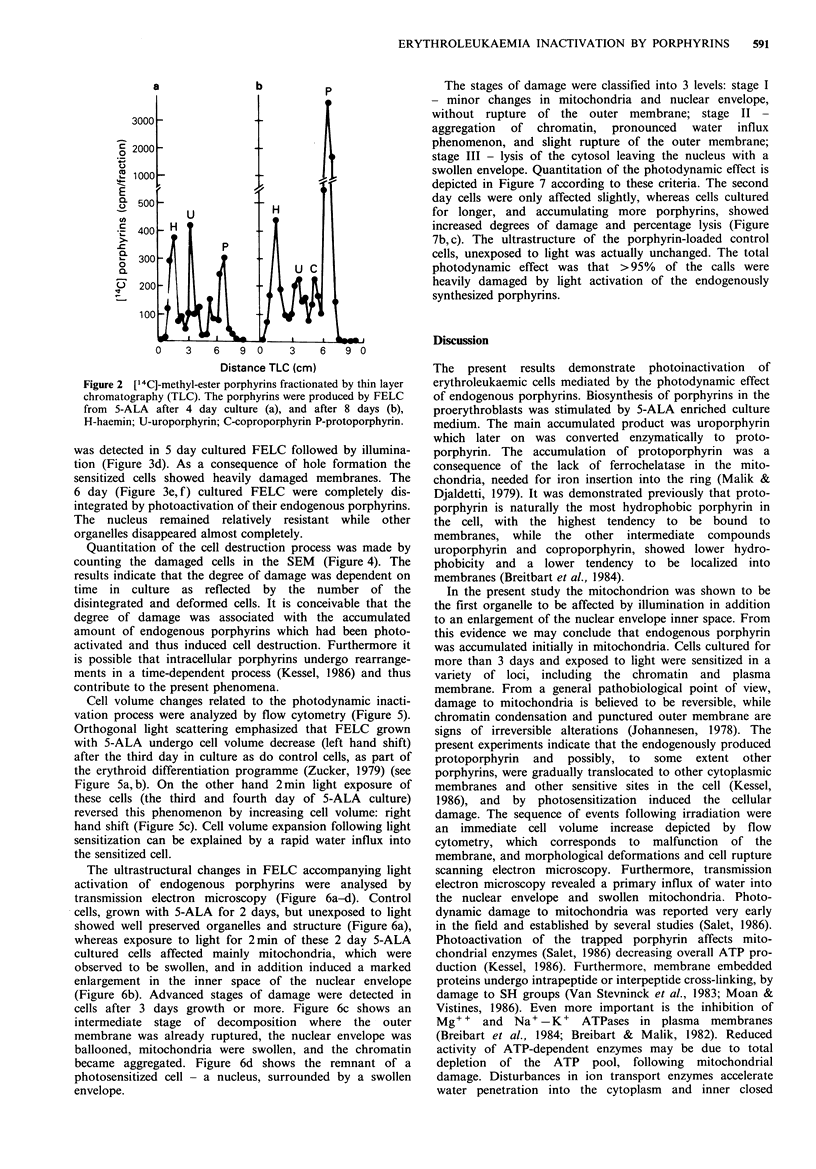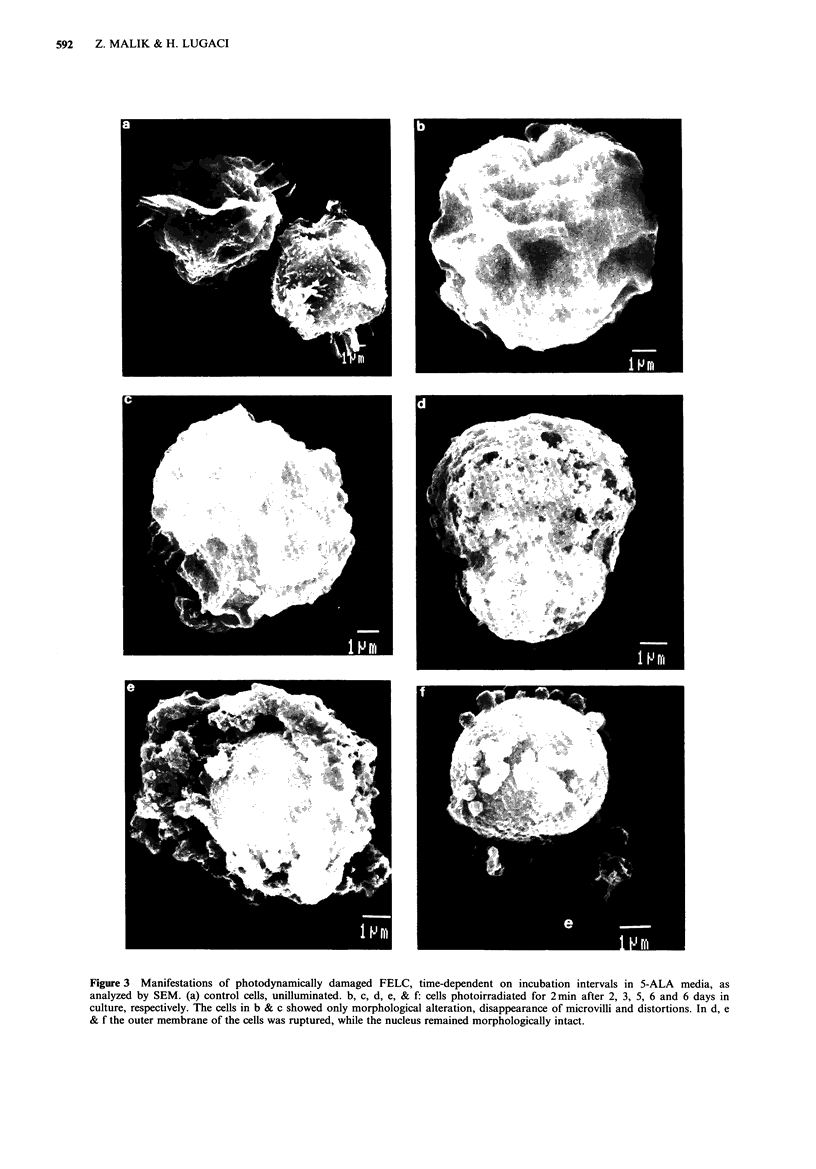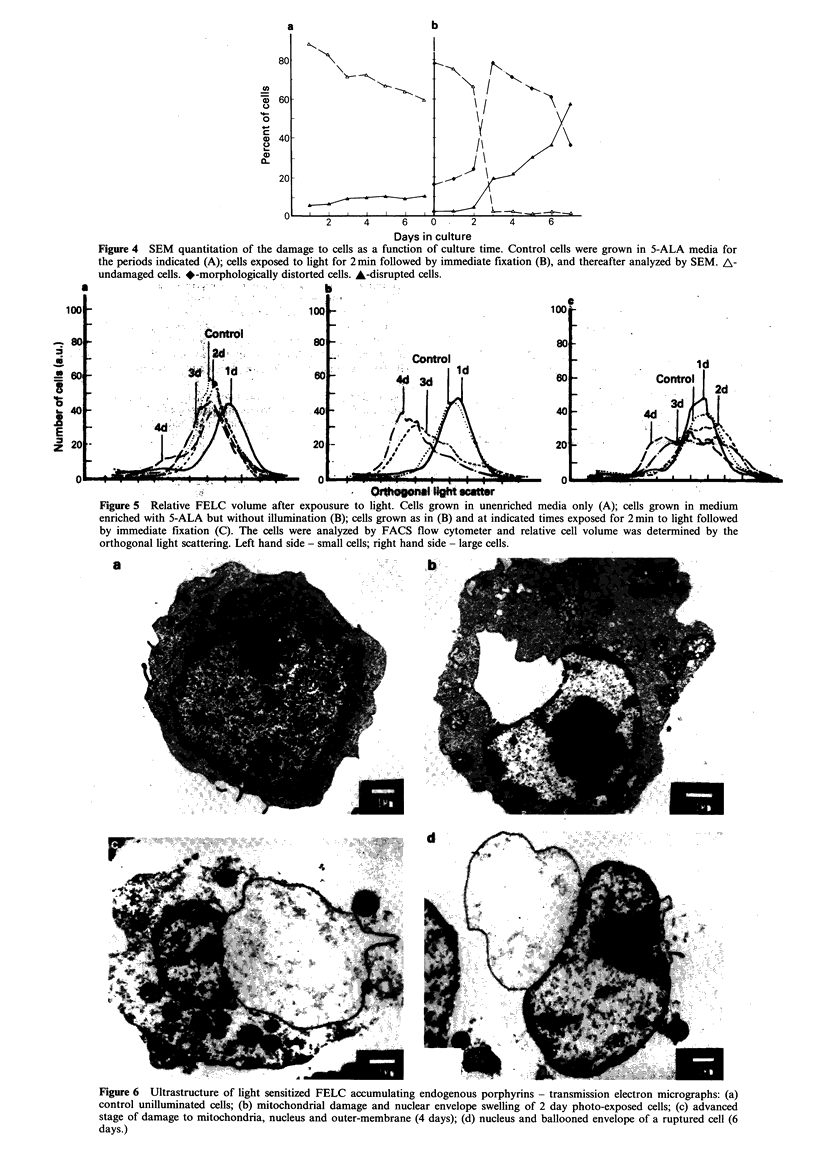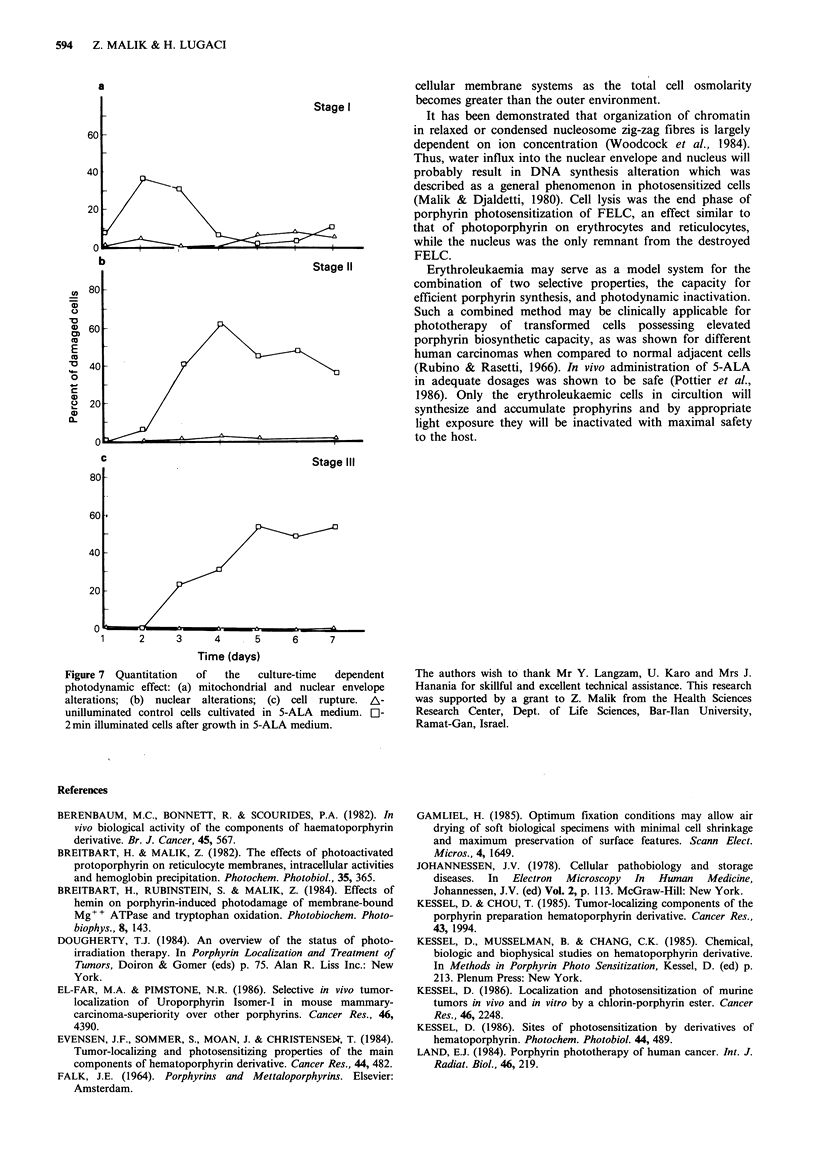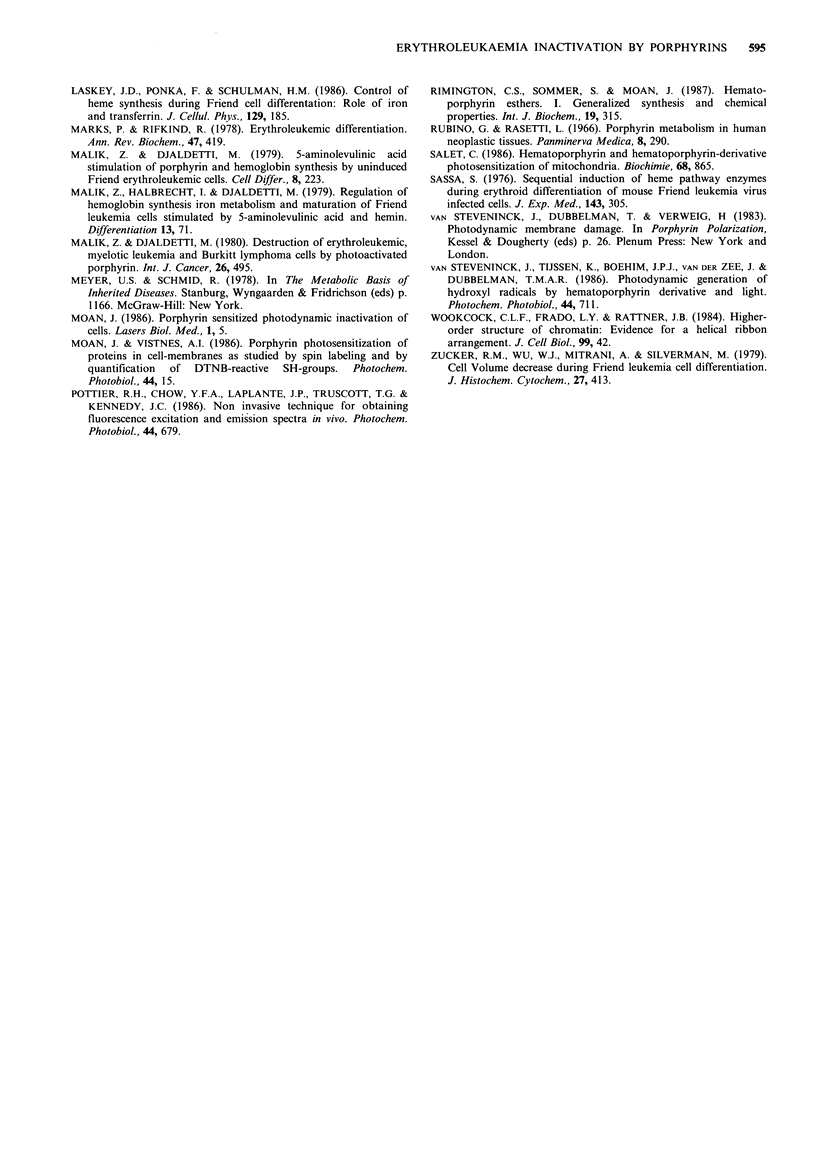# Destruction of erythroleukaemic cells by photoactivation of endogenous porphyrins.

**DOI:** 10.1038/bjc.1987.246

**Published:** 1987-11

**Authors:** Z. Malik, H. Lugaci

**Affiliations:** Department of Life Sciences, Bar-Ilan University, Ramat-Gan, Israel.

## Abstract

**Images:**


*Br. J. Cancer* (1987) **56**, 589–595 © The Macmillan Press Ltd., 1987

### Destruction of erythroleukaemic cells by photoactivation of endogenous porphyrins

Z. Malik & H. Lugaci

*Health Sciences Research Center, Department of Life Sciences, Bar-Ilan University, Ramat-Gan 52100, Israel.*

**Summary** Selective destruction of Friend erythroleukaemic cells (FELC) was potentiated by stimulation of endogenous porphyrin synthesis followed by light sensitization. Endogenous porphyrin biosynthesis in FELC was induced by supplementation of 5-amino levulinic acid (5-ALA) at a concentration of $5\times10^{-4}$ M. The main accumulated product, after 4 days culture, was uroporphyrin, while after 8 days culture the cells were loaded with protoporphyrin, up to $1.5\,\mu g\,10^{-7}$ cells. Photoirradiation of the cells for 2 min, accumulating endogenous porphyrins, induced cardinal deformations and cell disintegration in >95% of the cells, as examined by scanning electron microcopy (SEM). The photodynamic destruction effects were dependent on cultivation time with 5-ALA. Flow cytometry analysis showed an immediate expansion of cell volume subsequent to irradiation, presumably a consequence of water influx. Transmission electron microscopy (TEM) of photosensitized cells after different time intervals of culture in 5-ALA medium, revealed initial damage to mitochondria and water influx into the nuclear envelope, after 2 days. After 3–4 days in culture the water influx phenomenon was pronounced, chromatin condensation took place and slight rupture of the outer membrane was detected. Cells photosensitized after 5–6 days of culture were completely disintegrated leaving a nuclear remnant and an enormously swollen nuclear envelope. The culture time dependence of the process, showed an interrelationship between the photodynamic effect and porphyrin accumulation sites in cellular compartments. The study presents a specific method for erythroleukaemic cell inactivation.

Cancer therapy ideally should be based on high selectivity of the therapeutic agent for the transformed cells, low specificity for normal tissues and high cytotoxic efficiency to the target tumour. These requirements are partially fulfilled by haematoporphyrin derivative (HPD) phototherapy.

The method is based on the capability of porphyrins to be selectively localized in malignant tumours. Light activation of the localized porphyrin induces damage to mitochondria, cellular organelles, membranes, DNA, and specific proteins by singlet oxygen, produced under aerobic conditions and possibly by hydroxyl radicals (Reviewed by Moan, 1986; Kessel, *et al.*, 1985; & Van Steveninck *et al.*, 1986). Photodynamic therapy is based on administration of an HPD solution to the cancer patient and later on, illumination of the tumour by a 630 nm laser light beam. The treated area undergoes necrosis, with minimal changes in the surrounding tissues (Dougherty, 1984; Land, 1984; Berenbaum *et al.*, 1982).

Although HPD is quite an effective tumour localizer, the trend is to synthesize new porphyrin-compounds with higher tumour localizing capacities, and being chemically well defined, to overcome the main problem of HPD *viz.* its complex porphyrin composition and aggregation state (Kessel & Chou, 1985; Evensen *et al.*, 1984). Recently, a variety of new molecules with good tumour localization and sensitization properties were introduced into experimental systems, like haematoporphyrin di-ethers (Rimington *et al.*, 1987) and chlorin-porphyrin ester (Kessel, 1986). Uroporphyrin I was reevaluated mainly for diagnostic applications (El-Far & Pimstone, 1986).

On the other hand, it is well known that the natural protoporphyrin is an excellent photosensitizer, inducing haemolysis and light sensitivity of the skin in porphyric patients (Meyer & Schmid, 1978). It is a poor tumour localizer despite its high photo-activity potential on *in vitro* incubated cells (Malik & Djaldetti, 1980). In addition, protoporphyrin is biosynthesized in low amounts, by all tumour cells, as well as non-transformed tissues, while in specific transformed cells such as erythroleukaemia, it can be produced more efficiently (Malik & Djaldetti, 1979).

Correspondence: Z. Malik.
Received 9 March 1987; and in revised form, 18 June 1987.

Friend erythroleukaemic cells are proerythroblasts transformed by the Friend complex virus. These cells are capable of being induced to differentiate by the action of polar solvents and a variety of chemicals (Marks & Rifkind, 1978). In their proerythroblastic phase the enzymatic activity of porphyrin biosynthesis was found to be constitutive, except for the first enzyme, the ALA-synthase which is the rate limiting step of the pathway and is inducible (Sassa, 1976), and iron uptake from transferrin (Lasky *et al.*, 1986). Therefore, in order to induce porphyrin synthesis by erythroblasts, exogenous 5-ALA must be supplied to circumvent the first limiting enzyme (Malik *et al.*, 1979).

Mice injected with 5-ALA showed porphyrin production in the skin, an effect similar to that of 5-ALA in cultured Friend erythroleukaemic cells (Pottier *et al.*, 1986). From both these systems it can be concluded that the cellular concentration of porphyrin can be increased by exogenous addition of the precursor for porphyrins.

The purpose of the present study was to determine whether endogenous porphyrins produced from 5-ALA erythroleukaemic cells can serve as specific and selective cell destructive agents following photo-activation.

### Materials and methods

### *Cells and culture conditions*

Friend erythroleukaemia cells (FELC) line 745 subclone 21 were isolated by the soft agar technique. The cells were maintained in Dulbecco's modified Eagle's medium supplemented with 15% foetal calf serum (Gibco) in a humidified incubator enriched with 10% $CO_2$ at 37°C. The cells were subdivided twice a week by resuspension in fresh medium at a concentration of $\sim5\times10^5$ cells $ml^{-1}$. Cells were counted in a Neubauer chamber.

### *Porphyrin synthesis, esterification and chromatography*

FELC were grown in culture medium supplemented with cold ALA $5\times10^{-4}$ M and [4-$^{14}$C]ALA ($0.1\,\mu Ci\,ml^{-1}$) for 3 days. Cells ($10^8$) were harvested, washed twice in PBS (0.1 M, pH = 7.2), lysed and lyophylized. Porphyrins and haem were esterified by 5% $H_2SO_4$ in methanol for 120 min at 30°C.

The porphyrin methyl esters were transferred to chloroform and neutralized over 10% sodium bicarbonate. The chloroform solution was dried over anhydrous sodium sulphate. Thin layer chromatography was performed by application of chloroform solutions of porphyrins on to Merck 60 silica gel plates and developed in tanks containing benzene/ethyl acetate/methanol (85:14.4:1.5) for 30 min. Identification of porphyrin spots was achieved by running haemin, uroporphyrin, coproporphyrin and protoporphyrin methyl ester markers (Sigma).

The porphyrin bands were detected by their red fluorescence under ultraviolet light and photographed on Kodak Ektachrome film using a red filter on the camera lens (Falk, 1964). Haem was the non-fluorescenting spot accumulated just beyond the origin, in parallel with a haem marker.

*Quantitation of porphyrins*

Each porphyrin methyl ester band was scraped of the silica gel plate, put into a tube and eluted by shaking with 2 ml chloroform. Absorbance was read in a Gilford 240 spectrophotometer. Relevant Soret band wave lengths and extinction coefficients for haemin and porphyrin methyl esters were recorded as described by Falk (1964).

[$^{14}$C]Porphyrin methyl ester fractions were scraped off, extracted with chloroform and counted in Insta-Gel-Packard in a Tricarb counter (Packard). Elution efficiency of a protoporphyrin methyl ester standard was 92%.

*Photosensitization of cells*

FELC grown in 5-ALA enriched media were collected at indicated time intervals and washed in PBS (0.1 M, pH 7.2). The resuspended cells ($10^6\,ml^{-1}$) were irradiated for 2 min from 10 mm distance, using a 'black-light' source, delivering $10\,W\,m^{-2}$. The emission spectra of the light were in the region 320–450 nm, with a maximum at 380 nm. Less than 0.1°C rise in the medium was recorded after 2 min illumination. At the end of light exposure the cells were immediately collected and fixed by glutaraldehyde 2.5% in phosphate-buffer 0.1 M pH 7.2, at room temperature for 1 h and then processed for electron microscopy or flow cytometry.

*Preparation for transmition EM*

Cultures were fixed by 2.5% glutaraldehyde in phosphate buffer pH 7.2, post-fixed by osmium tetroxide 2%, embedded in Epon 812, thin sectioned by a LKB Ultratome III, and stained with uranyl acetate and lead citrate. The samples were examined by a Jeol 1200EX electron microscope.

*Preparation for SEM*

Cells were fixed by 2.5% glutaraldehyde in phosphate buffer pH 7.2, then washed in the same buffer, and post fixed by osmium tetroxide 2%. The third step of fixation was performed in a solution of tannic acid-guanidine hydrochloride (Gamliel, 1985). The triple fixed cells were dehydrated in graded alcohol solutions and then the alcohol was exchanged for Freon-112 by graded Freon solutions. The cells were air dried, gold coated and examined by a Jeol 840 SEM.

*Cell size determinations*

Cell samples at the end of each experiment were immediately fixed as described and subjected to flow cytometry analysis. Ten thousand cells were counted and analyzed in a Becton & Dickinson FACS 440 flow cytometer. Orthogonal scattered light determined cell volume in comparison to latex beads as standards. All samples were run on the same day and the collecting channels in each run were therefore identical.

### Results

Enrichment of the culture medium with 5-ALA stimulated porphyrin production in the undifferentiated proerythroblastic cells. Figure 1 shows the porphyrins produced in FELC after 8 days of incubation with 5-ALA. Protoporphyrin, coproporphyrin and uroporphyrin are the biosynthesised products. Control cells grown without 5-ALA contain only traces of these products, indicating that endogenous porphyrin synthesis by FELC is dependent on the internal pool of the precursor for the tetra-pyrrole rings in the cells.

Supplementation of exogenous 5-ALA plus [$^{14}$C]-ALA to the culture enabled quantitation of the relative amounts of each porphyrin produced (Figure 2). Thin layer chromatography followed by radioactivity determination of the fractions showed that after 4 days of incubation uroporphyrin was the main product followed by protoporphyrin, while after 8 days protoporphyrin was produced and accumulated to a very high level. The total amount of protoporphyrin was more than 5 times higher than all other porphyrins together. Spectrophotometric determination of the recovered protoporphyrin indicated an amount of $1.5\,\mu g\ 10^{-7}$ cells after 8 days in culture. It is conceivable that both uroporphyrin and protoporphyrin may act as photosensitizers in these cells. It should be emphasized that haemin, iron-protoporphyrin, was produced in the cells, besides the other metal-free porphyrins.

The unique metabolic capacity of these erythroid cells to synthesize and accumulate porphyrins was used for their selective inactivation. FELC were grown for different time intervals in 5-ALA medium, illuminated for 2 min and fixed for EM. Figure 3a–f depicts the process of erythroleukaemic cell destruction by photoactivation of endogenous porphyrins as analysed by SEM. Figure 3a shows control unilluminated cells grown for 3 days; the cells possessed microvilli and a ruffled surface. Figure 3b,c depicts the initial shape alterations induced by light exposure of 2–3 day cultured cells. These consisted of outer deformations and irregularities without direct damage to membrane integrity. Photodynamic rupture of the outer membrane

**Figure 1** Thin layer chromatography of methyl-ester-porphyrins from 8 day cultured FELC in 5-ALA enriched medium. Numbers on left indicate the numbers of carboxyl groups on tetrapyrrole ring.

**Figure 2** [$^{14}$C]-methyl-ester porphyrins fractionated by thin layer chromatography (TLC). The porphyrins were produced by FELC from 5-ALA after 4 day culture (a), and after 8 days (b), H-haemin; U-uroporphyrin; C-coproporphyrin P-protoporphyrin.

was detected in 5 day cultured FELC followed by illumination (Figure 3d). As a consequence of hole formation the sensitized cells showed heavily damaged membranes. The 6 day (Figure 3e, f) cultured FELC were completely disintegrated by photoactivation of their endogenous porphyrins. The nucleus remained relatively resistant while other organelles disappeared almost completely.

Quantitation of the cell destruction process was made by counting the damaged cells in the SEM (Figure 4). The results indicate that the degree of damage was dependent on time in culture as reflected by the number of the disintegrated and deformed cells. It is conceivable that the degree of damage was associated with the accumulated amount of endogenous porphyrins which had been photoactivated and thus induced cell destruction. Furthermore it is possible that intracellular porphyrins undergo rearrangements in a time-dependent process (Kessel, 1986) and thus contribute to the present phenomena.

Cell volume changes related to the photodynamic inactivation process were analyzed by flow cytometry (Figure 5). Orthogonal light scattering emphasized that FELC grown with 5-ALA undergo cell volume decrease (left hand shift) after the third day in culture as do control cells, as part of the erythroid differentiation programme (Zucker, 1979) (see Figure 5a, b). On the other hand 2 min light exposure of these cells (the third and fourth day of 5-ALA culture) reversed this phenomenon by increasing cell volume: right hand shift (Figure 5c). Cell volume expansion following light sensitization can be explained by a rapid water influx into the sensitized cell.

The ultrastructural changes in FELC accompanying light activation of endogenous porphyrins were analysed by transmission electron microscopy (Figure 6a–d). Control cells, grown with 5-ALA for 2 days, but unexposed to light showed well preserved organelles and structure (Figure 6a), whereas exposure to light for 2 min of these 2 day 5-ALA cultured cells affected mainly mitochondria, which were observed to be swollen, and in addition induced a marked enlargement in the inner space of the nuclear envelope (Figure 6b). Advanced stages of damage were detected in cells after 3 days growth or more. Figure 6c shows an intermediate stage of decomposition where the outer membrane was already ruptured, the nuclear envelope was ballooned, mitochondria were swollen, and the chromatin became aggregated. Figure 6d shows the remnant of a photosensitized cell – a nucleus, surrounded by a swollen envelope.

The stages of damage were classified into 3 levels: stage I – minor changes in mitochondria and nuclear envelope, without rupture of the outer membrane; stage II – aggregation of chromatin, pronounced water influx phenomenon, and slight rupture of the outer membrane; stage III – lysis of the cytosol leaving the nucleus with a swollen envelope. Quantitation of the photodynamic effect is depicted in Figure 7 according to these criteria. The second day cells were only affected slightly, whereas cells cultured for longer, and accumulating more porphyrins, showed increased degrees of damage and percentage lysis (Figure 7b, c). The ultrastructure of the porphyrin-loaded control cells, unexposed to light was actually unchanged. The total photodynamic effect was that >95% of the calls were heavily damaged by light activation of the endogenously synthesized porphyrins.

### Discussion

The present results demonstrate photoinactivation of erythroleukaemic cells mediated by the photodynamic effect of endogenous porphyrins. Biosynthesis of porphyrins in the proerythroblasts was stimulated by 5-ALA enriched culture medium. The main accumulated product was uroporphyrin which later on was converted enzymatically to protoporphyrin. The accumulation of protoporphyrin was a consequence of the lack of ferrochelatase in the mitochondria, needed for iron insertion into the ring (Malik & Djaldetti, 1979). It was demonstrated previously that protoporphyrin is naturally the most hydrophobic porphyrin in the cell, with the highest tendency to be bound to membranes, while the other intermediate compounds uroporphyrin and coproporphyrin, showed lower hydrophobicity and a lower tendency to be localized into membranes (Breitbart *et al.*, 1984).

In the present study the mitochondrion was shown to be the first organelle to be affected by illumination in addition to an enlargement of the nuclear envelope inner space. From this evidence we may conclude that endogenous porphyrin was accumulated initially in mitochondria. Cells cultured for more than 3 days and exposed to light were sensitized in a variety of loci, including the chromatin and plasma membrane. From a general pathobiological point of view, damage to mitochondria is believed to be reversible, while chromatin condensation and punctured outer membrane are signs of irreversible alterations (Johannesen, 1978). The present experiments indicate that the endogenously produced protoporphyrin and possibly, to some extent other porphyrins, were gradually translocated to other cytoplasmic membranes and other sensitive sites in the cell (Kessel, 1986), and by photosensitization induced the cellular damage. The sequence of events following irradiation were an immediate cell volume increase depicted by flow cytometry, which corresponds to malfunction of the membrane, and morphological deformations and cell rupture scanning electron microscopy. Furthermore, transmission electron microscopy revealed a primary influx of water into the nuclear envelope and swollen mitochondria. Photodynamic damage to mitochondria was reported very early in the field and established by several studies (Salet, 1986). Photoactivation of the trapped porphyrin affects mitochondrial enzymes (Salet, 1986) decreasing overall ATP production (Kessel, 1986). Furthermore, membrane embedded proteins undergo intrapeptide or interpeptide cross-linking, by damage to SH groups (Van Stevninck *et al.*, 1983; Moan & Vistines, 1986). Even more important is the inhibition of $Mg^{++}$ and $Na^{+}-K^{+}$ ATPases in plasma membranes (Breibart *et al.*, 1984; Breibart & Malik, 1982). Reduced activity of ATP-dependent enzymes may be due to total depletion of the ATP pool, following mitochondrial damage. Disturbances in ion transport enzymes accelerate water penetration into the cytoplasm and inner closed

**Figure 3** Manifestations of photodynamically damaged FELC, time-dependent on incubation intervals in 5-ALA media, as analyzed by SEM. (a) control cells, unilluminated. b, c, d, e, & f: cells photoirradiated for 2 min after 2, 3, 5, 6 and 6 days in culture, respectively. The cells in b & c showed only morphological alteration, disappearance of microvilli and distortions. In d, e & f the outer membrane of the cells was ruptured, while the nucleus remained morphologically intact.

**Figure 4** SEM quantitation of the damage to cells as a function of culture time. Control cells were grown in 5-ALA media for the periods indicated (A); cells exposed to light for 2 min followed by immediate fixation (B), and thereafter analyzed by SEM. △-undamaged cells. ◆-morphologically distorted cells. ▲-disrupted cells.

**Figure 5** Relative FELC volume after expousure to light. Cells grown in unenriched media only (A); cells grown in medium enriched with 5-ALA but without illumination (B); cells grown as in (B) and at indicated times exposed for 2 min to light followed by immediate fixation (C). The cells were analyzed by FACS flow cytometer and relative cell volume was determined by the orthogonal light scattering. Left hand side – small cells; right hand side – large cells.

**Figure 6** Ultrastructure of light sensitized FELC accumulating endogenous porphyrins – transmission electron micrographs: (a) control unilluminated cells; (b) mitochondrial damage and nuclear envelope swelling of 2 day photo-exposed cells; (c) advanced stage of damage to mitochondria, nucleus and outer-membrane (4 days); (d) nucleus and ballooned envelope of a ruptured cell (6 days.)

**Figure 7** Quantitation of the culture-time dependent photodynamic effect: (a) mitochondrial and nuclear envelope alterations; (b) nuclear alterations; (c) cell rupture. △-unilluminated control cells cultivated in 5-ALA medium. □-2 min illuminated cells after growth in 5-ALA medium.

cellular membrane systems as the total cell osmolarity becomes greater than the outer environment.

It has been demonstrated that organization of chromatin in relaxed or condensed nucleosome zig-zag fibres is largely dependent on ion concentration (Woodcock *et al.*, 1984). Thus, water influx into the nuclear envelope and nucleus will probably result in DNA synthesis alteration which was described as a general phenomenon in photosensitized cells (Malik & Djaldetti, 1980). Cell lysis was the end phase of porphyrin photosensitization of FELC, an effect similar to that of photoporphyrin on erythrocytes and reticulocytes, while the nucleus was the only remnant from the destroyed FELC.

Erythroleukaemia may serve as a model system for the combination of two selective properties, the capacity for efficient porphyrin synthesis, and photodynamic inactivation. Such a combined method may be clinically applicable for phototherapy of transformed cells possessing elevated porphyrin biosynthetic capacity, as was shown for different human carcinomas when compared to normal adjacent cells (Rubino & Rasetti, 1966). *In vivo* administration of 5-ALA in adequate dosages was shown to be safe (Pottier *et al.*, 1986). Only the erythroleukaemic cells in circultion will synthesize and accumulate prophyrins and by appropriate light exposure they will be inactivated with maximal safety to the host.

The authors wish to thank Mr Y. Langzam, U. Karo and Mrs J. Hanania for skillful and excellent technical assistance. This research was supported by a grant to Z. Malik from the Health Sciences Research Center, Dept. of Life Sciences, Bar-Ilan University, Ramat-Gan, Israel.

### References

BERENBAUM, M.C., BONNETT, R. & SCOURIDES, P.A. (1982). *In vivo* biological activity of the components of haematoporphyrin derivative. *Br. J. Cancer*, **45**, 567.

BREITBART, H. & MALIK, Z. (1982). The effects of photoactivated protoporphyrin on reticulocyte membranes, intracellular activities and hemoglobin precipitation. *Photochem. Photobiol.*, **35**, 365.

BREITBART, H., RUBINSTEIN, S. & MALIK, Z. (1984). Effects of hemin on porphyrin-induced photodamage of membrane-bound $Mg^{++}$ ATPase and tryptophan oxidation. *Photobiochem. Photobiophys.*, **8**, 143.

DOUGHERTY, T.J. (1984). An overview of the status of photoirradiation therapy. In *Porphyrin Localization and Treatment of Tumors*, Doiron & Gomer (eds) p. 75. Alan R. Liss Inc.: New York.

EL-FAR, M.A. & PIMSTONE, N.R. (1986). Selective *in vivo* tumorlocalization of Uroporphyrin Isomer-I in mouse mammary-carcinoma-superiority over other porphyrins. *Cancer Res.*, **46**, 4390.

EVENSEN, J.F., SOMMER, S., MOAN, J. & CHRISTENSEN, T. (1984). Tumor-localizing and photosensitizing properties of the main components of hematoporphyrin derivative. *Cancer Res.*, **44**, 482.

FALK, J.E. (1964). *Porphyrins and Mettaloporphyrins*. Elsevier: Amsterdam.

GAMLIEL, H. (1985). Optimum fixation conditions may allow air drying of soft biological specimens with minimal cell shrinkage and maximum preservation of surface features. *Scann Elect. Micros.*, **4**, 1649.

JOHANNESSEN, J.V. (1978). Cellular pathobiology and storage diseases. In *Electron Microscopy In Human Medicine*, Johannessen, J.V. (ed) **Vol. 2**, p. 113. McGraw-Hill: New York.

KESSEL, D. & CHOU, T. (1985). Tumor-localizing components of the porphyrin preparation hematoporphyrin derivative. *Cancer Res.*, **43**, 1994.

KESSEL, D., MUSSELMAN, B. & CHANG, C.K. (1985). Chemical, biologic and biophysical studies on hematoporphyrin derivative. In *Methods in Porphyrin Photo Sensitization*, Kessel, D. (ed) p. 213. Plenum Press: New York.

KESSEL, D. (1986). Localization and photosensitization of murine tumors *in vivo* and *in vitro* by a chlorin-porphyrin ester. *Cancer Res.*, **46**, 2248.

KESSEL, D. (1986). Sites of photosensitization by derivatives of hematoporphyrin. *Photochem. Photobiol.* **44**, 489.

LAND, E.J. (1984). Porphyrin phototherapy of human cancer. *Int. J. Radiat. Biol.*, **46**, 219.

LASKEY, J.D., PONKA, F. & SCHULMAN, H.M. (1986). Control of heme synthesis during Friend cell differentation: Role of iron and transferrin. *J. Cellul. Phys.*, **129,** 185.

MARKS, P. & RIFKIND, R. (1978). Erythroleukemic differentiation. *Ann. Rev. Biochem.*, **47,** 419.

MALIK, Z. & DJALDETTI, M. (1979). 5-aminolevulinic acid stimulation of porphyrin and hemoglobin synthesis by uninduced Friend erythroleukemic cells. *Cell Differ.*, **8,** 223.

MALIK, Z., HALBRECHT, I. & DJALDETTI, M. (1979). Regulation of hemoglobin synthesis iron metabolism and maturation of Friend leukemia cells stimulated by 5-aminolevulinic acid and hemin. *Differentiation* **13,** 71.

MALIK, Z. & DJALDETTI, M. (1980). Destruction of erythroleukemic, myelotic leukemia and Burkitt lymphoma cells by photoactivated porphyrin. *Int. J. Cancer*, **26,** 495.

MEYER, U.S. & SCHMID, R. (1978). In *The Metabolic Basis of Inherited Diseases*. Stanburg, Wyngaarden & Fridrichson (eds) p. 1166. McGraw-Hill: New York.

MOAN, J. (1986). Porphyrin sensitized photodynamic inactivation of cells. *Lasers Biol. Med.*, **1,** 5.

MOAN, J. & VISTNES, A.I. (1986). Porphyrin photosensitization of proteins in cell-membranes as studied by spin labeling and by quantification of DTNB-reactive SH-groups. *Photochem. Photobiol.*, **44,** 15.

POTTIER, R.H., CHOW, Y.F.A., LAPLANTE, J.P., TRUSCOTT, T.G. & KENNEDY, J.C. (1986). Non invasive technique for obtaining fluorescence excitation and emission spectra *in vivo*. *Photochem. Photobiol.*, **44,** 679.

RIMINGTON, C.S., SOMMER, S. & MOAN, J. (1987). Hematoporphyrin esthers. I. Generalized synthesis and chemical properties. *Int. J. Biochem.*, **19,** 315.

RUBINO, G. & RASETTI, L. (1966). Porphyrin metabolism in human neoplastic tissues. *Panminerva Medica*, **8,** 290.

SALET, C. (1986). Hematoporphyrin and hematoporphyrin-derivative photosensitization of mitochondria. *Biochimie*, **68,** 865.

SASSA, S. (1976). Sequential induction of heme pathway enzymes during erythroid differentiation of mouse Friend leukemia virus infected cells. *J. Exp. Med.*, **143,** 305.

VAN STEVENINCK, J., DUBBELMAN, T. & VERWEIG, H (1983). Photodynamic membrane damage. In *Porphyrin Polarization*, Kessel & Dougherty (eds) p. 26. Plenum Press: New York and London.

VAN STEVENINCK, J., TIJSSEN, K., BOEHIM, J.P.J., VAN DER ZEE, J. & DUBBELMAN, T.M.A.R. (1986). Photodynamic generation of hydroxyl radicals by hematoporphyrin derivative and light. *Photochem. Photobiol.*, **44,** 711.

WOOKCOCK, C.L.F., FRADO, L.Y. & RATTNER, J.B. (1984). Higher-order structure of chromatin: Evidence for a helical ribbon arrangement. *J. Cell Biol.*, **99,** 42.

ZUCKER, R.M., WU, W.J., MITRANI, A. & SILVERMAN, M. (1979). Cell Volume decrease during Friend leukemia cell differentiation. *J. Histochem. Cytochem.*, **27,** 413.